# Depletion of b-series ganglioside prevents limb length discrepancy after growth plate injury

**DOI:** 10.1186/s12891-024-07704-7

**Published:** 2024-07-20

**Authors:** Yoshiaki Hosokawa, Masatake Matsuoka, Yuko Sakai, Ryuichi Fukuda, Keizumi Matsugasaki, Kentaro Homan, Jun-ichi Furukawa, Tomohiro Onodera, Norimasa Iwasaki

**Affiliations:** 1 Department of Orthopaedic Surgery, Faculty of Medicine, Graduate School of Medicine, Hokkaido University, North 15 West 7, Kita-Ku, Sapporo, Hokkaido 060-8638 Japan; 2 Institute for Glyco-core Research (iGCORE), Nagoya University, Nagoya, 464-8601 Japan

**Keywords:** Growth plate, Glycosphingolipid, Ganglioside, Orthopedic surgery, Growth plate cartilage

## Abstract

**Introduction:**

Growth plate damage in long bones often results in progressive skeletal growth imbalance and deformity, leading to significant physical problems. Gangliosides, key glycosphingolipids in cartilage, are notably abundant in articular cartilage and regulate chondrocyte homeostasis. This suggests their significant roles in regulating growth plate cartilage repair.

**Methods:**

Chondrocytes from 3 to 5 day-old C57BL/6 mice underwent glycoblotting and mass spectrometry. Based on the results of the glycoblotting analysis, we employed GD3 synthase knockout mice (GD3-/-), which lack b-series gangliosides. In 3-week-old mice, physeal injuries were induced in the left tibiae, with right tibiae sham operated. Tibiae were analyzed at 5 weeks postoperatively for length and micro-CT for growth plate height and bone volume at injury sites. Tibial shortening ratio and bone mineral density were measured by micro-CT.

**Results:**

Glycoblotting analysis indicated that b-series gangliosides were the most prevalent in physeal chondrocytes among ganglioside series. At 3 weeks, GD3-/- exhibited reduced tibial shortening (14.7 ± 0.2 mm) compared to WT (15.0 ± 0.1 mm, *P* = 0.03). By 5 weeks, the tibial lengths in GD3-/- (16.0 ± 0.4 mm) closely aligned with sham-operated lengths (*P* = 0.70). Micro-CT showed delayed physeal bridge formation in GD3-/-, with bone volume measuring 168.9 ± 5.8 HU at 3 weeks (WT: 180.2 ± 3.2 HU, *P* = 0.09), but normalizing by 5 weeks.

**Conclusion:**

This study highlights that GD3 synthase knockout mice inhibit physeal bridge formation after growth plate injury, proposing a new non-invasive approach for treating skeletal growth disorders.

## Introduction

The epiphyseal plate, a cartilaginous layer found at the ends of long bones in juveniles, is crucial for the vertical development of bones [1–4]. Damage to this structure can lead to the formation of a bony bar at the site of the injury, which is a primary cause of limb deformity. This bony bar impedes normal skeletal growth, causing imbalances and deformities that result in significant physical complications. Currently, the primary intervention to address severe imbalances and deformities, often stemming from the formation of a bony bar, involves surgical procedures such as the excision of the bony bar and corrective surgery for limb deformities. However, these surgical options are not only highly invasive but also impose a great deal of stress on the patient and their family.

Glycans are integral to regulating metabolic pathways, including those in cartilage tissue, where Glycosphingolipids (GSLs)—a category of glycolipids prevalent in the plasma membranes of vertebrates—play a vital role [5, 6]. A systemic deficiency of GSLs has been linked to fatal outcomes in embryos, while a targeted deficiency in Type II collagen precipitates the acceleration of osteoarthritic cartilage degeneration [7, 8]. This evidence suggests a critical role for GSLs in maintaining chondrocyte equilibrium. Delving into GSLs’ regulatory functions on chondrocytes could unveil novel treatments for musculoskeletal disorders.

Particularly, gangliosides—a subgroup of GSLs, which use GM3 as a starting point for synthesis—are notably abundant in cartilage, with studies showing a 40% reduction in total ganglioside content in osteoarthritic cartilage [9, 10]. This reduction hints at a significant role in cartilage metabolism and differentiation, with ganglioside depletion observed to enhance articular cartilage integrity [11]. Thus, gangliosides emerge as potential agents in cartilage regeneration therapies. While specific GSLs downstream of GM3 in the ganglioside series have been considered in treatment strategies for osteoarthritis, their role in the cartilage repair process has not been thoroughly examined [12, 13]. In addition, given their vital function in joint cartilage repair, GSLs are also presumed to be significant in the repair processes of the growth plate cartilage, although their exact role remains to be elucidated.

As a result, our study hypothesizes that gangliosides may exert a critical influence on the repair of growth plate cartilage through their impact on cell differentiation and metabolic processes. Our investigation commenced with the identification of the most abundant specific GSLs situated downstream of GM3 within the ganglioside sequence in physeal cartilage. Subsequently, we examined the reparative function of these gangliosides in growth plate cartilage by utilizing mice with a deficiency in ganglioside series synthase.

## Methods

### Experimental animals

The Institutional Animal Care and Use Committee of Hokkaido university granted approval for all procedures (20–0098 and 24–0074). Mice lacking GD3 synthase (GD3-/-) were bred and developed following the methodology outlined in prior research [14]. GD3-/- were originally a mix of C57BL/6 and 129/Sv strains, but were subsequently backcrossed these mice to the C57BL/6 strain, resulting in a C57BL/6 background [15]. We acquired C57BL/6 mice, serving as wild-type (WT) controls, from Japan SLC, Inc. These mice were maintained in an environment with controlled temperature and humidity, subjected to a 12-hour light/dark cycle. Their diet consisted of standard rodent feed, adhering to our institution’s guidelines for the ethical treatment and care of laboratory animals.

### Euthanasia and sacrifice methods

Prior to sacrifice, the animals were anaesthetised to ensure they were unconscious and did not experience pain. The anaesthetic agent used was ketamine, administered at a dosage of 100 mg/kg via intraperitoneal injection, and xylazine, administered at a dosage of 10 mg/kg via intraperitoneal injection. Once fully anaesthetised, euthanasia was performed using cervical dislocation.

### Operative procedure for physeal cartilage injury in mice

**Fig. 1:**
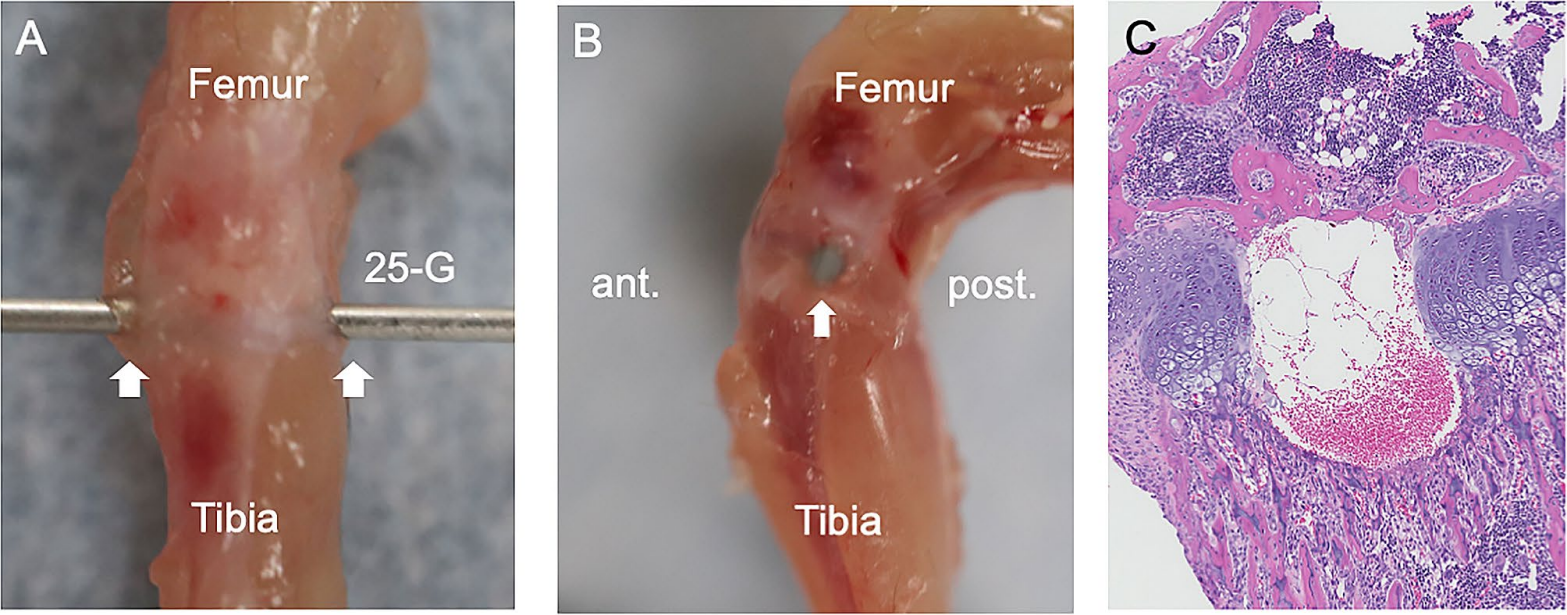
3-week-old C57BL/6 mice were subjected to a growth plate injury model. After anesthesia, a ~ 10 mm incision was made in the skin over the proximal tibia using a scalpel, exposing the tibiae. The growth plate was pierced laterally to medially using a sterilized 25-G needle, and the incision was closed with sutures. (A, B) Cadaveric demonstration of the procedure, **(A)** front view, **(B)** lateral view. **(C)** Histological analysis of the tibial growth plate post-injury, stained with hematoxylin and eosin

In 3-week-old female WT and GD3-/-, full-thickness injuries to the physeal region were induced (10 mice per group). A microsurgical scalpel was then utilized to create an incision approximately 0.5 cm in length, exposing the tibiae. The entire proximal tibial growth plate was methodically penetrated in a lateral to medial direction with a 25-G needle, as depicted in Fig. 1 [16]. These microsurgical procedures were performed under a surgical microscope (SZX16; Olympus, Tokyo, Japan). After irrigation with normal saline to remove debris, the skin was sutured in separate layers. In the experimental setup, the injury group had physeal injuries created in the left knee, while the control group underwent a sham operation on the right knee of the same animals. This sham operation involved only making an incision in the skin to expose the growth plate, with no further manipulation. Following general anesthesia, the hind limbs were aseptically prepared. Postoperatively, mice were warmed until recovery from anesthesia was confirmed. Tibiae were harvested and analyzed at 3 or 5 weeks postoperatively.

### Macroscopic evaluation

At either 3 or 5 weeks after surgery, both tibiae were collected from each mouse (10 mice per time point). The entire tibia was meticulously separated, then gathered and photographed alongside a millimeter-scale indicator. Subsequently, the specimens were preserved in 10% formalin (WAKO, Tokyo, Japan) for histological examination. The tibial length, extending from the inferior articular surface to the tibial plateau, was measured using Image J software (version 1.53c; National Institutes of Health). We calculated the tibia-shortening ratio (referred to as the ‘drop-ratio’), which represents the percentage difference in tibia length between the sham-operated and operated limbs, normalized to the length of the sham-operated tibia.

### Micro-Computed Tomography (Micro-CT) analysis

Micro-computed tomography (micro-CT) was employed to assess the formation of bony bars within the damaged physeal area, as well as to measure the bone mineral density (BMD) in the subchondral region [17–19]. The tibiae were scanned using micro-CT (70 kVp; 200 µA; integration time of 300 ms) to achieve an isotropic voxel size of 15.6 μm, utilizing the VivaCT80 system from Scanco. A specific volume of interest (VOI) was delineated manually in the sagittal plane. This VOI encompassed a standardized area within the physeal region, measuring 2.5 mm in width and extending through 160 slices (approximately 2.5 mm in depth), thereby including the entire bony bar formed within the injured physis. Global thresholding was applied to these image stacks, and the extent of bony bar formation was quantified, denoted as the bone volume/total volume (BV/TV) ratio as BMD. Additionally, BMD was evaluated in the subchondral zone, 40 slices beneath the growth plate. BMD were computed using the Bone J plugin for ImageJ software (version 1.53c; National Institutes of Health). The BMD ratio was calculated as the BMD value on the operated side relative to the BMD on the sham-operated side.

### Histological processing

For each designated time point, the tibiae were separated from the joints and preserved in 10% formalin sourced from Wako, Tokyo, Japan. This was followed by decalcification using ethylenediaminetetraacetic acid (EDTA). During the paraffin embedding process, special care was taken to align the tibial axis perpendicularly to the embedding surface. Subsequently, midsagittal sections were prepared at intervals of 5 micrometers. These sections were then subjected to staining using hematoxylin and eosin (H.E.) for histological examination.

### Isolation of physeal cartilage

Murine physeal chondrocytes were extracted from the knee joints of neonatal mice, following established protocols [20–22]. Culturing was performed using Dulbecco’s Modified Eagle’s Medium (DMEM, WAKO) supplemented with 10% fetal bovine serum (FBS, NICHIREI) and 1% antibiotic solution (WAKO).

### Quantification of Ganglioside-glycans in the physeal chondrocytes by mass spectrometry

Gangliosides were extracted from physeal chondrocyte pellets using the chloroform-methanol method, while GSL-glycans were liberated through endoglycoceramidase digestion, following established procedures [11, 12]. The resulting intact ganglioside-glycans were subsequently subjected to glycoblotting and analyzed using matrix-assisted laser desorption ionization-time-of-flight/time-of-flight mass spectrometry [23].

### Statistics

Statistical analyses of the data were conducted using JMP Pro 16.0 statistical software (SAS Institute Inc., Cary, NC, USA). Although, it has been reported that the growth of GD3-/- is comparable to WT [13], in a clinical context, it is appropriate to use the contralateral side as the reference for evaluating the shortening/deformation of the injured tibia. Therefore, an unpaired t-test was used to compare the operated tibia and the contralateral sham-operated tibia. In addition, we standardized the measurements using the drop ratio, defined as the percentage difference in tibia length between sham operated and operated tibiae relative to the length of the sham operated tibia. Consequently, the statistical analysis of tibial length in the growth plate injury group was paired with the respective sham-operated tibia, and we compared the shortening rate, standardized by the drop ratio between WT and GD3-/- at each time point. The results are expressed as the mean ± standard error of the mean (SEM), and significance was considered at *p* < 0.05.

## Results

### Quantification of ganglioside-glycans in physeal chondrocytes revealed that b-series gangliosides are most abundantly present in the epiphyseal cartilage

**Fig. 2:**
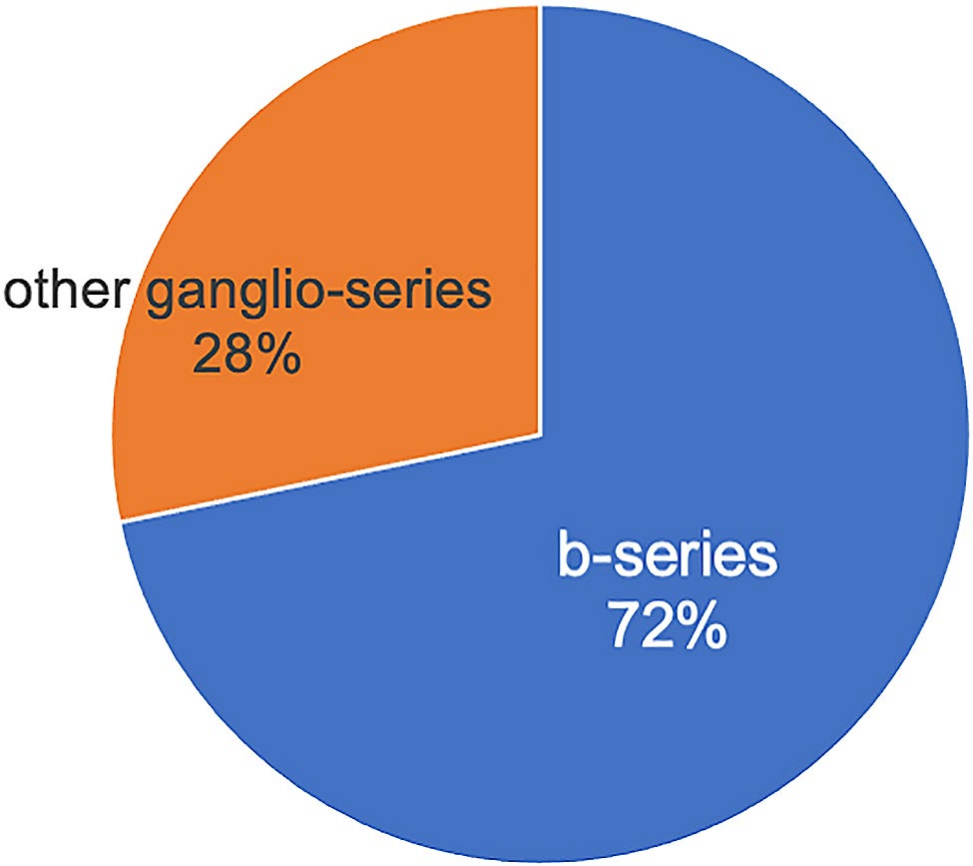
Quantitative analysis of Ganglioside-glycans in cultured mouse physeal chondrocytes (*n* = 3)

Initially, to explore potential therapeutic targets downstream of GM3, the starting point of ganglioside synthesis, we performed ganglioside quantification using mass spectrometry. Interestingly, glycoblotting analysis revealed that b-series gangliosides, originating from GD3 synthase, were the most abundant gangliosides in physeal chondrocytes among the ganglioside series (Fig. 2). Therefore, in subsequent experiments, we employed GD3-/-, which lacked b-series gangliosides.

### Depletion of b-series gangliosides inhibited impaired bone growth

**Fig. 3:**
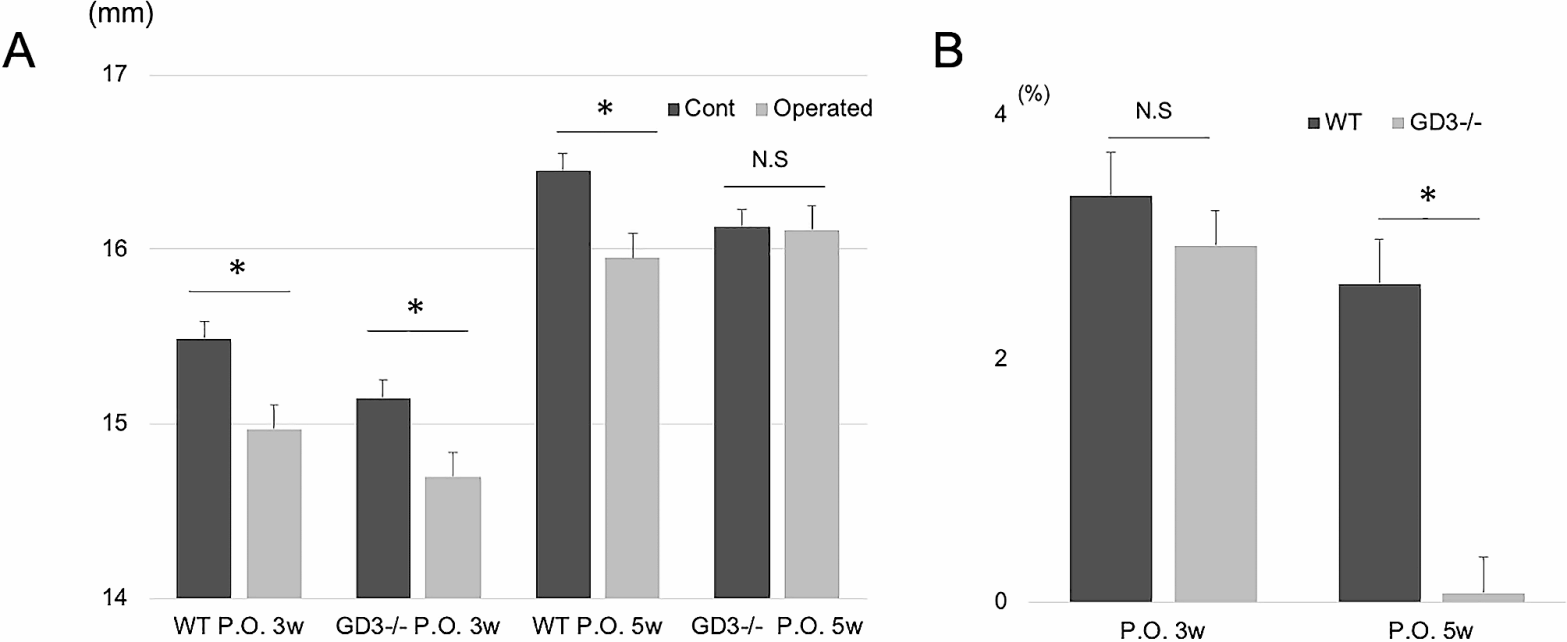
Macroscopic analysis of tibiae (*n* = 10). **(A)** Comparison of tibial lengths in each group post-surgery. **(B)** Comparison of drop ratio, defined as the percentage decrease in tibial length between the sham-operated and operated tibiae, relative to the length of the sham-operated tibia. *P < 0.05

At 3 weeks post-surgery, the tibial length on the operated side was significantly shorter compared to the sham-operated side in both WT and GD3-/- (WT: mean ± SEM, 15.0 ± 0.1 mm in operated vs. 15.5 ± 0.1 mm in sham, *P* = 0.01; GD3-/-: 14.7 ± 0.2 mm in operated vs. 15.2 ± 0.1 mm in sham, *P* = 0.03; Fig. 3A). By 5 weeks postoperatively, the length of the operated tibia had significantly shortened in WT compared to the sham-operated tibia (mean ± SEM, 16.0 ± 0.5 mm in operated vs. 16.5 ± 0.4 mm in sham, *P* = 0.04; Fig. 3A). In contrast, there was no significant difference in tibial length between operated and sham-operated limbs in GD3-/- (mean ± SEM, 16.0 ± 0.4 mm in operated vs. 16.0 ± 0.3 mm in sham, *P* = 0.70; Fig. 3A).

The drop ratio, defined as the percentage difference in tibia length between sham-operated and operated tibiae relative to the length of the sham-operated tibia, was nearly identical between WT and GD3-/- at 3 weeks postoperatively (mean ± SEM, 3.3 ± 0.7% in WT vs. 2.9 ± 1.1% in GD3-/-, *P* = 0.69; Fig. 3B). However, by 5 weeks postoperatively, the drop ratio in GD3-/- had significantly decreased compared to WT (mean ± SEM, 3.1 ± 1.2% in WT vs. 0.3 ± 1.4% in GD3-/-, *p* < 0.01; Fig. 2B). These results suggest that GD3-/- exhibited reduced impairment in bone growth at 5 weeks post-surgery.

### Micro-CT evaluation and histology: b-series deficiency shows hindered formation of the physeal bridge

**Fig. 4:**
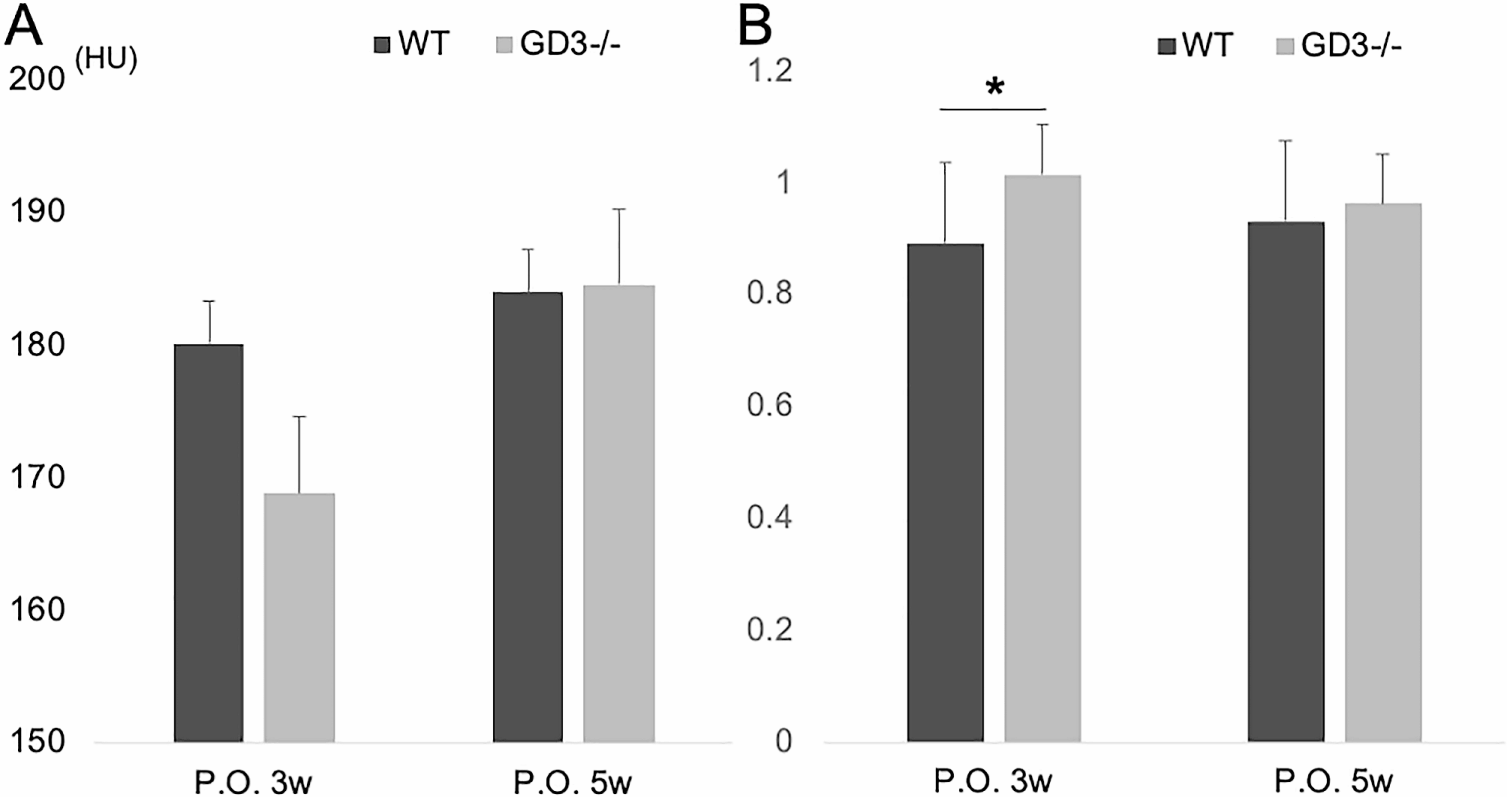
Micro-CT analysis (*n* = 10): **(A)** Physeal bar’s CT value comparison. **(B)** BMD ratio comparison, defined as the operated side’s BMD divided by the sham-operated side’s BMD. *P < 0.05

We next investigated the reason why the leg length discrepancy observed in GD3-/- at 3 weeks post-surgery is resolved by 5 weeks post-surgery. This was assessed using micro-CT. Interestingly, the bone volume of the physeal bridge in GD3-/- tended to decrease at 3 weeks postoperatively (mean ± SEM, 180.2 ± 3.2 HU in WT vs. 168.9 ± 5.8 HU in GD3-/-, *P* = 0.09; Fig. 4A). However, by 5 weeks postoperatively, the bone volume in GD3-/- was almost equal to that in WT (mean ± SEM, 184.0 ± 11.8 HU in WT vs. 184.5 ± 12.0 HU in GD3-/-, *P* = 0.91; Fig. 4A). These results indicate that GD3-/- does not inhibit physeal bar formation but rather delays it.

Further analysis was conducted on the secondary spongiosa, crucial for bone formation along the longitudinal axis of long bones. At 3 weeks postoperatively, the BMD ratio in WT was significantly reduced compared to GD3-/- (mean ± SEM, 0.9 ± 0.1 in WT vs. 1.0 ± 0.1 in GD3-/-, *P* < 0.01; Fig. 4B). Conversely, at 5 weeks postoperatively, the BMD ratios in WT and GD3-/- were almost equivalent (mean ± SEM, 0.9 ± 0.1 in WT vs. 1.0 ± 0.1 in GD3-/-, *P* = 0.58; Fig. 4B).

**Fig. 5:**
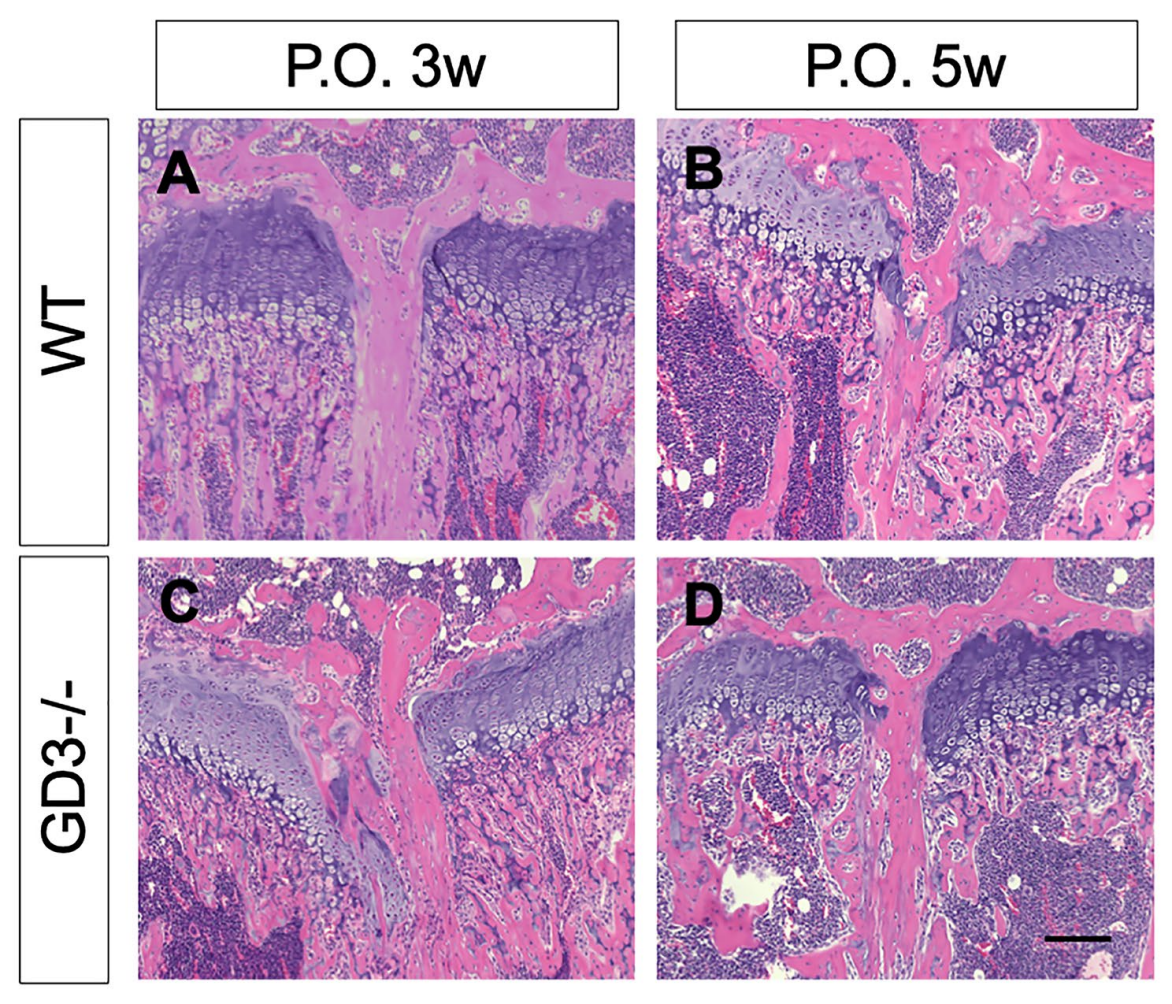
Histological analysis of physeal bridge formation at various time points post-injury: The histology reveals variations in the bony composition of the physeal bridge with **(A and B)** or without **(C and D)** GD3 synthase. Scale bar: 0.2 mm

The BMD ratio findings in GD3-/- suggest that the delayed formation of the physeal bar at 3 weeks post-surgery may preserve the activity of the secondary spongiosa, thus maintaining growth along the longitudinal axis of the long bones. H.E. staining revealed differences in physeal bridge formation at the injury site. WT mice exhibited a denser, more continuous bony bar at 3 weeks (Fig. 5A), whereas the physeal bridge in GD3-/- appeared sparse, unstable, and discontinuous, with some empty areas (Fig. 5C). Matured physeal bar formation was confirmed at 5 weeks in both WT and GD3-/- (Fig. 5B and D). The histological analysis results corroborated the findings obtained from micro-CT.

## Discussion

This study has revealed that the absence of b-series gangliosides in GD3 synthase-knockout mice leads to an interesting phenomenon where bone growth impairment, commonly seen post physeal injury, is significantly mitigated. The delayed formation of the physeal bridge in the absence of these gangliosides suggests a possible regulatory role that extends beyond their structural functions in cell membranes. The use of GD3-/- has allowed us to observe the potential reparative advantages in growth plate injury repair, challenging existing paradigms of ganglioside functions in chondrocyte biology and opening avenues for non-invasive therapeutic approaches. Additionally, GD3-/- were repeatedly utilized to investigate the regulation of cartilage/bone metabolism [13, 24]. Interestingly, GD3-/- show growth and development comparable to wild-type mice, but they have been reported to prevent bone loss in aging mice [24]. Combined with our research findings, the development of GD3 synthase inhibitor/antagonist could be a breakthrough in cartilage/bone metabolism.

It is established that mice reach maturation between 8 and 10 weeks of age, however, the longitudinal growth of the tibia peaks during the 5 to 8 weeks age range [25]. In our study, the delayed formation of the physeal bar in GD3-/- appeared to mitigate the inhibition of longitudinal tibial growth during this critical 3 to 6 weeks period, which in turn, may contribute to the suppression of growth disorders observed at 8 weeks of age. It is well-understood that chondrocytes in the growth plate undergo a chondrocyte maturation sequence, starting from the resting zone, progressing through the proliferating zone and the hypertrophic zone, and ultimately contributing to the formation of the secondary spongiosa [26–28]. At the 3-week postoperative mark, we observed that the bone density of the secondary spongiosa was reduced on the operated side in WT, whereas in GD3-/-, it was comparable to WT. The delay in physeal bar formation in GD3-/- may prevent disruption of the chondrocyte maturation sequence, allowing for the formation of the secondary spongiosa and, consequently, may prevent growth disorders.

The presence of nerve fibers has been identified in the perichondrium, cartilage canals, epiphyseal plate, and at the interface between the perichondrium and articular cartilage, as well as within the matrix surrounding chondrocytes during development [29]. In addition, the potential neuroprotective effects and neurite outgrowth-promoting activities of GM3 have been previously reported [30]. Considering these previous studies, it is suggested that nerve fibers may play a role in the healing process after growth plate injury. However, our study did not investigate this aspect. Future studies are needed to explore this potential role.

In this study, it became apparent that GD3-/- does not impede physeal bar formation; instead, it results in a delay in its development. Previous studies have reported varying behaviors of ganglioside series during osteogenic differentiation, with some showing decreased expression [31]. As our study focused on in vivo analysis of physeal bar formation, a detailed ganglioside analysis using glycoblotting was not able to conduct. However, it is likely that the absence of b-series gangliosides resulted in the lack of specific gangliosides that regulate osteogenesis, thereby delaying physeal bar formation. In previous study, we reported that in GM3 synthase knockout mice, which lack all gangliosides, chondrocyte hypertrophy is suppressed during the articular cartilage repair process [11]. In current study, we identified a particularly important subgroup of gangliosides synthesized by GD3 synthase, known as b-series gangliosides, using the glycoblotting method. Our results showed delayed bone bridge formation at the site of growth plate injury, which we believe is likely due to the inhibition of the chondrocyte hypertrophy process. On the other hand, previous research has shown that downstream ganglioside series can act in a coordinated and complementary manner [13]. Therefore, even with the deficiency of b-series gangliosides, the effects might be compensated by other ganglioside series, leading to a phenotype of delayed, rather than inhibited, physeal bar formation. A more detailed analysis of the role of ganglioside series in physeal bar formation warrants future study.

While our study provides important insights into the potential role of b-series gangliosides in growth plate repair, several limitations must be acknowledged. First, the use of GD3 synthase-knockout mice may not accurately represent the human condition, as the physiology of murine models can significantly differ from that of humans, particularly in complex processes such as bone growth and repair. Second, the compensatory biological mechanisms that may be activated due to the absence of b-series gangliosides in knockout mice could lead to an overestimation or underestimation of their role in human chondrocyte function and injury repair. Third, the study’s scope did not include long-term outcomes and the potential systemic effects of ganglioside depletion, leaving unanswered questions about the durability of the observed effects and overall homeostasis. These limitations underscore the necessity for cautious interpretation of the results and the need for further research to validate the findings in a clinical context.

In conclusion, our findings suggest that b-series gangliosides play a more complex role in the growth plate cartilage repair process than previously recognized. The modulation of ganglioside pathways could represent a novel therapeutic target for enhancing growth plate repair and mitigating growth impairments in a non-invasive manner, thereby offering an alternative to the surgical interventions currently in practice. Further research is needed to understand the molecular mechanisms underlying these observations and to translate these findings into clinical applications.

## Data Availability

The dataset supporting the conclusions of this article is available at our institution contacting the corresponding author.
